# B7-H4 expression and its role in interleukin-2/interferon treatment of clear cell renal cell carcinoma

**DOI:** 10.3892/ol.2014.1961

**Published:** 2014-03-11

**Authors:** YIPENG XU, SHAOXING ZHU, MEI SONG, WEIHUI LIU, CHENGYI LIU, YONGSHENG LI, MIN WANG

**Affiliations:** 1 Department of Urology, Zhejiang Cancer Hospital, Hangzhou, Zhejiang 310022, P.R. China; 2 Department of Ultrasonography, Zhejiang Cancer Hospital, Hangzhou, Zhejiang 310022, P.R. China; 3 Department of Urology, Fujian Medical University Second Affiliated Hospital, Quanzhou, Fujian 362000, P.R. China; 4 Department of Urology, Lu’an Affiliated Hospital of Anhui Medical University, Lu’an, Anhui 237005, P.R. China; 5 Department of Urology, The Union Hospital of Fujian Medical University, Fuzhou, Fujian 350001, P.R. China; 6 Institute of Ophthalmology, Shandong Academy of Medical Sciences, Qingdao, Shandong 266071, P.R. China

**Keywords:** B7-H4, clear cell renal cell carcinoma, cytokines, tumor immunity

## Abstract

The immunological mechanism mediated by T cells is the main therapeutic target in the treatment of renal cell carcinoma (RCC) with interleukin (IL)-2 and interferon (IFN)-α. The aim of the present study was to evaluate the role of B7-H4 in the IL-2, IFN-α and IFN-γ treatment of clear cell RCC (ccRCC). A total of 154 paraffin-embedded ccRCC tissues were studied using immunohistochemistry, which subsequently indicated that positive B7-H4 expression is associated with adverse clinical features in ccRCC. The effects of IL-2, IFN-α and IFN-γ on B7-H4 expression in a ccRCC cell line were evaluated at the mRNA and protein levels. In addition, the effect of B7-H4 on the killing activity of T cells was detected. B7-H4 expression was identified to be upregulated by IL-2, IFN-α and IFN-γ, of which, IFN-γ was the most capable. Additionally, blocking of B7-H4/B7-H4 ligand interactions may rescue the killing activity of T cells. Altogether, the observations of the current study showed that the immune escape pathway induced by B7-H4 may be one of the most important reasons for the low efficacy of IL-2 and IFN-α and the inability to observe the efficacy of IFN-γ in mRCC. This indicates that B7-H4 may be used as a new molecular biology marker to select treatment options for patients with ccRCC.

## Introduction

Renal cell carcinoma (RCC) is one of the most common types of malignant tumor of the human urinary system. To date, the benefit of conventional therapies for RCC, including surgical, radiological and chemotherapeutic approaches, is limited. Treatment with interferon (IFN) and interleukin (IL)-2 remains the main immunotherapy method for RCC, with the exception of surgery, but only ~10% of advanced RCC patients respond to cytokine-based immunotherapy (1,2). Therefore, more effective potential and combined therapies must be found. New targeted therapy for RCC may initiate a new avenue for cancer treatment, and targeted therapy depends on the evaluation of target gene status.

B7-H4, also called B7x/B7s/VTCN1, is the newest B7 superfamily member identified as an inhibitory modulator of the T-cell response. Combined with its receptor, B7-H4 may inhibit the proliferation and cytokine production of CD4^+^ and CD8^+^ T cells. The blocking of B7-H4/B7-H4 ligand interactions may restore antitumor T-cell responses to ovarian cancer cells (3). Previous studies have reported that the B7-H4^+^ status is an independent predictor of poor prognosis in multivariate analysis (4–9). To date, few previous studies have analyzed the potential contributions of B7-H4 to tumoral immune escape and therapeutic targeting in RCC.

Herein, we present evidence for the potential contributions of B7-H4 to tumoral immune escape in ccRCC, which indicates that B7-H4 may be used as a new biological molecular marker for select treatment options in patients with ccRCC.

## Materials and methods

### Cell culture, antibodies and cytokines

The cell line, 786-0, was purchased from the Cell Bank at the Chinese Academy of Sciences (Beijing, China) and was cultured according to the manufacturer’s instructions. The anti-B7-H4 antibody (Ab) was purchased from R&D Systems (Minneapolis, MN, USA). Other Abs were purchased from Bioss (Beijing, China) and the cytokines (IL-2, IFN-α and IFN-γ) were purchased from Xiamen Amoytop Biotech Co., Ltd. (Xiamen, China).

### RCC tissue

A total of 154 specimens of RCC tissue were collected from RCC patients undergoing radical nephrectomy in the Department of Urology, Zhejiang Cancer Hospital (Hangzhou, China). The final staging, grading and histological diagnosis were based on the pathology report. Ethics approval was obtained from the local Institution Review Board committee.

### Immunohistochemistry (IHC) to tissue microarray (TMA) and 786-0 cells

IHC was performed using a polyclonal B7-H4 Ab at a dilution of 1:400. Antigen retrieval was performed by heating the slides for 7 min in 10 mM citric acid buffer. The TMA consisted of cores from 154 patients with clear cell RCC (ccRCC). IHC was analyzed independently by two pathologists, and positive IHC was determined when ≥5% of the cells showed B7-H4 staining. The 786-0 cells were fixed by 4% paraformaldehyde and directly incubated with B7-H4 Ab at a dilution of 1:400. The remaining procedures were performed as described for the TMA.

### Cell proliferation assay

Cell proliferation was quantitated by a Cell Counting Kit-8 (CCK-8) assay to generate a growth inhibition ratio following stimulation with IL-2, IFN-α and IFN-γ for 24 h. 786-0 cells were seeded at 6,000 cells per well in a 96-well plate and incubated for 24 h. The culture fluid, containing IL-2, IFN-α and IFN-γ at the concentrations of 0, 100, 250, 500, 1,000, 2,000, 4,000 and 8,000 U/ml, was then added into each well and incubated for 24 h. Next, 10 μl CCK-8 was added into each well and incubated for 2 h. Each well was read at 450 nm using a spectrophotometer (Eppendorf, Hamburg, Germany).

### ELISA to cell culture supernatant

The protein of B7-H4 was diluted to 100, 50, 25, 10, 2, 0.5 and 0.1 ng/ml to be used as a standard. Samples were collected following centrifugation at 1,200 × g. In total, 40 μl sample, 10 μl anti-B7-H4 Ab and 50 μl streptomycin-horseradish peroxidase were added into the ELISA kit and then incubated for 1 h at 37°C. Following washing three times with phosphate-buffered saline (PBS), 100 μl chromogenic agent was added to each well. In addition, 50 μl stop buffer was added to each well following incubation for 15 min at 37°C. The plates were then read at 450 nm using a spectrophotometer. The minimum detectable concentration was determined to be >0.1 ng/ml.

### Reverse transcription (RT)-polymerase chain reaction (PCR)

Total RNA was extracted from the 786-0 cells using TRIzol reagent (Invitrogen Life Technologies, Carlsbad, CA, USA) following stimulation with IL-2, IFN-α and IFN-γ (1,000 U/ml) for 24 h. A total of 2 μg RNA was reverse-transcribed using avian myeloblastosis virus reverse transcription XL (Toyobo Co. Ltd, Shanghai, China) for 90 min at 42°C in the presence of oligo(dT) primer (Fermentas, Waltham, MA, USA). PCR was performed using Taq polymerase. The primer sequences (Ying Wei Chuang, Guangzhou, China) used were as follows: B7-H4 forward, 5′-CACTCATCATTGGCTTTGGTATTTCAG-3′ and reverse, 5′-CGACAGCTCATCTTTGCCTTCTTTG-3′; and actin forward, 5′-AGCGGGAAATCGTGCGTGAC-3′ and reverse, 5′-ACTCCTGCTTGCTGATCCATATC-3′. PCR was performed for 35 cycles, which consisted of a pre-soak for 5 min at 94°C, denaturing for 30 sec at 94°C, annealing for 30 sec at 56°C and extension for 30 sec at 72°C. Following completion of the cycle, the amplified products were electrophoresed through a 1% agarose gel and stained with ethidium bromide. Images were captured under an ultraviolet light transilluminator (Syngene Co., Cambrisge, UK).

### Flow cytometry

The surface expression of B7-H4 on the 786-0 cell line following stimulation with IL-2, IFN-α and IFN-γ was quantified by flow cytometry on a fluorescence-activated cell sorter (FACs). For each analysis, 10,000 cells were evaluated. For detecting intracellular B7-H4 expression, the 786-0 cells were pre-permeabilized with permeabilization buffer for 10 min. Following washing twice with PBS, the cells were further fixed by fixation buffer (4% paraformaldehyde) and then B7-H4 monoclonal Abs (mAbs) were added. Following extreme washing with PBS, B7-H4 expression was further detected by FACs. The FACs results were analyzed using CELLQuest™ software (BD Biosciences, Franklin Lakes, NJ, USA).

### Functional cytotoxic assays with blocking B7-H4 mAb

Lymphoblastoid cell lines were established from mononuclear cells collected from the peripheral blood of healthy donors by Ficoll-Hypaque centrifugation. All individuals provided written informed consent. The cells were incubated with concanavalin A (1, 2, 4 and 8 μg/ml) for 48 h, and CCK-8 was performed to detect the lymphocyte proliferation rate of the T cells. Functional assays were performed by incubation of the T cells for 48 h, in the absence or presence of isotypic control (purified mouse IgG1 κ) or B7-H4 blocking mAb. The cytotoxic effect on the T cells was evaluated by CCK-8, which identifies apoptotic cells.

### Statistical analyses

SPSS version 13.0 (SPSS, Inc., Chicago, IL, USA) and Microsoft Excel 2003 (Microsoft Corporation, Redmond, WA, USA) were used for the statistical analyses. P<0.05 was considered to indicate a statistically significant difference.

## Results

### Increased B7-H4 expression is associated with adverse clinical features

**Figure 1:**
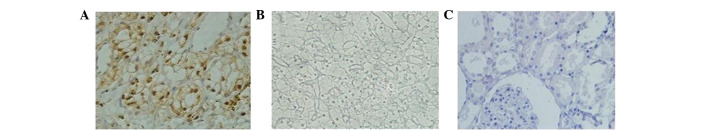
B7-H4 expression in normal kidney and RCC tissues. (A) Representative RCC tumor specimen with marked membranous tumor cell B7-H4 immunohistochemical staining. (B) RCC tumor specimen with negative tumor cell B7-H4 endothelial staining. (C) Normal tumor-adjacent kidney specimen without B7-H4 staining (magnification, ×200). RCC, renal cell carcinoma.

In total, 91 (59.09%) patient specimens exhibited positive tumor B7-H4 staining (Fig. 1A). A comparison of clinical features by tumor B7-H4 expression is shown in Table I. Positive tumor B7-H4 expression was associated with adverse clinical features, including tumor-node-metastasis and clinical stages.

### IL-2, IFN-α and IFN-γ may upregulate B7-H4 expression in 786-0 cells

**Figure 2:**
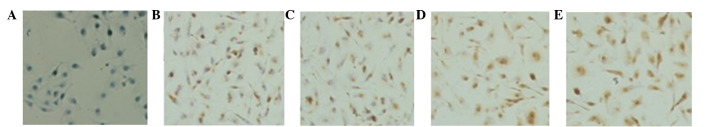
B7-H4 protein expression in 786-0 cells. (A) Negative control; (B) unstimulated; (C) stimulated by interleukin-2; (D) stimulated by interferon (IFN)-α; and (E) stimulated by IFN-γ (magnification, ×200).

**Figure 3:**
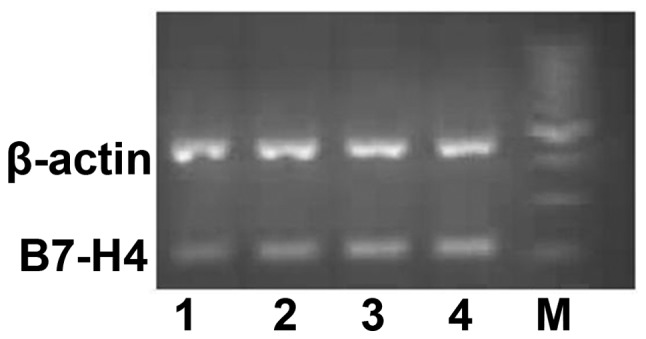
B7-H4 mRNA expression in 786-0 cells: 1, unstimulated; 2, stimulated by interleukin-2; 3, stimulated by interferon (IFN)-α; 4, stimulated by IFN-γ; and M, DNA marker.

The CCK-8 assay, a proliferation assay that is directly proportional to the number of live cells in culture, was used as an independent measure of the proliferation in the IL-2-, IFN-α- and IFN-γ-treated 786-0 cells. These results supported the fact that IL-2, IFN-α and IFN-γ may inhibit the proliferative activity of 786-0 cells and exhibit a significant dose-effect correlation. The maximum drug concentration of a 1% cellular proliferation inhibition rate was 1,000 U/ml. IL-2, IFN-α and IFN-γ were applied at this concentration to stimulate the 786-0 cells in order to study the effect of IL-2, IFN-α and IFN-γ on B7-H4 expression in ccRCC cells. The RT-PCR and IHC results showed that the protein (Fig. 2)and mRNA (Fig. 3) expression of B7-H4 may be upregulated by IL-2, IFN-α and IFN-γ, of which, IFN-γ was the most capable.

**Figure 4:**
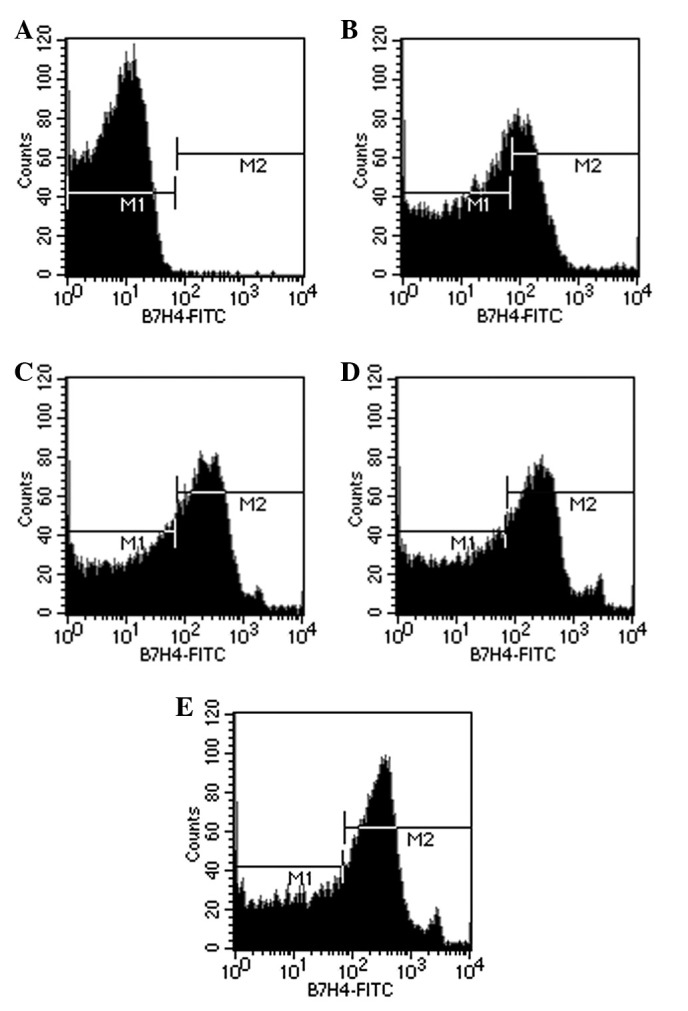
B7-H4 positive expression in 786-0 cells. (A) Control. (B) Unstimulated. (C) Stimulated with IL-2. (D) Stimulated with IFN-α. (E) Stimulated with IFN-γ.

The ELISA assay was used to detect the quantitative expression of soluble B7-H4 (sB7-H4) in the IL-2-, IFN-α- and IFN-γ-treated 786-0 cells. The sB7-H4 expression was detected in the unstimulated 786-0 cells at a concentration of 34.42±1.69 ng/ml. Following stimulation with IL-2, IFN-α and IFN-γ for 24 h, the concentrations increased to 44.89±0.97 ng/ml, 46.74±2.25 ng/ml and 47.31±1.12 ng/ml, respectively, in which the differences were statistically significant (P<0.05). Flow cytometry (Fig. 4), which supplied quantified results of the positive expression of B7-H4 in the 786-0 cells, revealed similar results. The positive expression rate of B7-H4 in the unstimulated 786-0 cells was 30.45±0.96%. Following stimulation with IL-2, IFN-α and IFN-γ for 24h, the positive expression rates became 44.89±0.94, 46.41±0.55 and 54.18±1.42%, respectively, which were significantly different compared with the unstimulated cells (P<0.05). The 786-0 cells stimulated by IFN-γ were the most capable, and the positive expression rate was significantly different compared with the other three groups (P<0.05). However, no significant difference was identified between the cells stimulated by IL-2 and IFN-α (P=0.235).

### B7-H4 is a negative regulator of T-cell cytotoxicity

To determine the role of B7-H4 in T-cell responses, different ratios of effector to target cells (30:1, 20:1 and 10:1) were applied. CCK-8 was used to detect the cytotoxicity of the T cells. The results showed that masking B7-H4 with a specific blocking Ab increased the T-cell killing of the 786-0 cells (P<0.05). With the increase of the concentration of effector cells, the cytotoxicity also increased significantly (P<0.05). This identified the inhibitory role of B7-H4 in T cell-dependent cytotoxicity, as in other tumor cell models (3).

## Discussion

RCC is a typical immunogenic tumor frequently harboring high levels of tumor-infiltrating T lymphocytes and occasionally exhibiting spontaneous regression of metastases following primary tumor removal (10–12). As it is refractory to radiation and chemotherapy, immunotherapy, including the use of IFN-α and IL-2, is the main treatment choice for mRCC without surgery. However, in previous studies, the efficacy of IL-2 and IFN-α has been extremely low in mRCC and it has not been possible to observe the efficacy of IFN-γ (1,2).

The costimulatory B7 family members not only provide positive signals to stimulate T-cell activation, but also deliver negative signals to inhibit T-cell responses. Identified in 2003, B7-H4 represents the newest member of the B7 family of costimulatory ligands (13–15). Despite widespread B7-H4 mRNA expression in various human tissues, the lack of immunohistochemical staining of B7-H4 in the majority of normal human tissues indicates that the expression of B7-H4 is relatively restricted (6). B7-H4 is a type I transmembrane protein, and expression may be detected in various types of human cancer tissues, including breast (4), ovarian (5), pancreatic (6) and lung (7) cancer, melanoma (8) and RCC (9). The expression of B7-H4 has been found to correlate with advanced stages, poor patient survival and tumor infiltration by T regulatory cells (16), which made it a candidate of choice for targeted therapy. Notably, in the present study, positive B7-H4 expression was associated with adverse clinical features in ccRCC, which indicated that B7-H4 may be a feasible candidate of choice for RCC-targeted therapy.

Tumor infiltrating lymphocytes may be a manifestation of antitumor immunity, but a more abundant infiltration of tumor tissue T cells has been associated with a shorter survival of the RCC patients (11), which indicated that there is a potential failure mechanism of T-cell immunity in RCC tissues. The immunological mechanism mediated by T cells is also the main therapeutic target in the IL-2 and IFN-α treatment of mRCC patients. The results of the present study demonstrated that IL-2, IFN-α and IFN-γ may upregulate the expression of B7-H4, which may inhibit the proliferation and cytokine production of CD4^+^ and CD8^+^ T cells. Additionally, IFN-γ was found to be the most capable for this, which indicated that the immune escape pathway induced by B7-H4 may be one of the most important reasons for the low efficacy of IL-2 and IFN-α and the inability to observe the efficacy of IFN-γ in mRCC treatment.

The current study further evaluated the functional ability to reverse T-cell inhibition mediated by the B7-H4 protein, which indicated that masking B7-H4 with a specific blocking Ab may increase the cytotoxicity of T cells in ccRCC. These results confirmed that B7-H4 is a regulatory molecule engaged in negative signaling that impacts anti-responses mediated by T cells in ccRCC, and also establishes a new paradigm for ccRCC cell eradication using B7-H4-based targeting. The study indicates that the blocking of B7-H4/B7-H4 ligand interactions may represent a feasible therapeutic strategy for ccRCC.

Targeting immune checkpoint molecules, such as CTLA4 and B7-H1, has elicited marked clinical responses, particularly in patients with pre-existing immune responses (17–19). Based on our studies, we propose that the blocking of B7-H4/B7-H4 ligand interactions may be used as a potential treatment for RCC patients. In addition, B7-H4 detection may be used to select the appropriate treatment for RCC patients. RCC patients whose TMA shows positive expression of B7-H4 must not select IFN-α/IL-2 treatment alone, since the T-cell-mediated antitumor responses must have been repressed by the B7-H4 mediated immune escape pathway. B7-H4 blocking treatment alone or combined with IFN-α/IL-2 may be more suitable for B7-H4^+^ patients.

## Figures and Tables

**Table I:** Clinical features of tumor B7-H4 expression.

Feature	B7-H4^−^ expression (n=63)	B7-H4^+^ expression (n=91)	χ^2^ (Fisher)	P-value
Gender				
Male	18	27	0.022	0.883
Female	45	64		
Age at surgery, years				
≥65	20	28	0.017	0.898
<65	43	63		
2009 primary tumor classification				
T_1_	52	52	13.291	0.004
T_2_	10	29		
T_3_	1	9		
T_4_	0	1		
Regional lymph node involvement				
N_x_/N_0_	63	87	4.282	0.039
N_1_/N_2_	0	4		
Distant metastases at nephrectomy				
M_0_	63	86	5.377	0.020
M_1_	0	5		
2009 TNM stage groupings				
I	50	53	9.583	0.022
II	11	25		
III	1	6		
IV	1	7		

TNM, tumor-node-metastasis.
